# Use of and access to oral and injectable contraceptives in Brazil

**DOI:** 10.1590/S1518-8787.2016050006176

**Published:** 2016-12-01

**Authors:** Mareni Rocha Farias, Silvana Nair Leite, Noemia Urruth Leão Tavares, Maria Auxiliadora Oliveira, Paulo Sergio Dourado Arrais, Andréa Dâmaso Bertoldi, Tatiane da Silva Dal Pizzol, Vera Lucia Luiza, Luiz Roberto Ramos, Sotero Serrate Mengue

**Affiliations:** I Departamento de Ciências Farmacêuticas. Centro de Ciências da Saúde. Universidade Federal de Santa Catarina. Florianópolis, SC, Brasil; II Departamento de Farmácia. Faculdade de Ciências da Saúde. Universidade de Brasília. Brasília, DF, Brasil; III Escola Nacional de Saúde Pública Sérgio Arouca. Fundação Oswaldo Cruz. Rio de Janeiro, RJ, Brasil; IV Departamento de Farmácia. Faculdade de Farmácia, Odontologia e Enfermagem. Universidade Federal do Ceará. Fortaleza, CE, Brasil; V Departamento de Medicina Social. Faculdade de Medicina. Universidade Federal de Pelotas. Pelotas, RS, Brasil; VI Departamento de Produção e Controle de Medicamentos. Faculdade de Farmácia. Universidade Federal do Rio Grande do Sul. Porto Alegre, RS, Brasil; VII Escola Paulista de Medicina. Universidade Federal de São Paulo. São Paulo, SP, Brasil; VIII Programa de Pós-Graduação em Epidemiologia. Faculdade de Medicina. Universidade Federal do Rio Grande do Sul. Porto Alegre, RS, Brasil

**Keywords:** Contraceptive Agents, supply & distribution, Contraceptives, Oral, supply & distribution, Health Services Accessibility, Socioeconomic Factors, Health Surveys

## Abstract

**OBJECTIVE:**

To analyze the prevalence of current use of oral and injectable contraceptives by Brazilian women, according to demographic and socioeconomic variables and issues related to access to those medicines.

**METHODS:**

A cross-sectional, population-based analytical study with probability sampling based on data from the *Pesquisa Nacional sobre Acesso, Utilização e Promoção do Uso Racional de Medicamentos* (PNAUM – National Survey on Access, Use and Promotion of Rational Use of Medicines), carried out between September 2013 and February 2014 in 20,404 Brazilian urban households. Prevalence was calculated based on reports from non-pregnant women aged 15-49 on the use of oral or injectable contraceptives. The independent variables were gender, age, level of education, socioeconomic class, Brazilian region and marital status. Also analyzed were access, means of payment, sources, and reported medicines. Statistical analyses considered 95% confidence intervals (95%CI) and Pearson Chi-square test to evaluate the statistical significance of differences between groups, considering a 5% significance level.

**RESULTS:**

Prevalence of use was 28.2% for oral contraceptives (OC) and 4.5% for injectable contraceptives (IC). The highest prevalence of oral contraceptives was in the South region (37.5%) and the lowest in the North region (15.7%). For injectable contraceptives there was no difference between regions. Access was higher for oral contraceptive users (90.7%) than injectable contraceptives users (81.2%), as was direct payment (OC 78.1%, IC 58.0%). Users who paid for contraceptives acquired them at retail pharmacies (OC 95.0% and IC 86.6%) and at *Farmácia Popular* (Popular Pharmacy Program) (OC 4.8% and IC 12.7%). Free of charge contraceptives were mostly obtained from the Brazilian Unified Health System – SUS (OC 86.7%; IC 96.0%). Free samples were reported by 10.4% of users who did not pay for oral contraceptives. Most of paying users did not try to obtain contraceptives from SUS. Monophasic combined oral contraceptives were the most frequently reported (71.6%) and low-level levonorgestrel + ethinylestradiol combination accounted for 38.7% of them. The most frequently reported medicines are included in the *Relação Nacional de Medicamentos Essenciais* (RENAME – National List of Essential Medicines.

**CONCLUSIONS:**

Most women aged 15 to 49 who reported using contraceptives had access to the medicine and use monophasic combined oral contraceptives of appropriate efficiency and safety purchased by direct payment, mainly from retail pharmacies.

## RESUMO

**OBJETIVO:**

Analisar a prevalência do uso atual de contraceptivos orais e injetáveis por mulheres brasileiras, segundo variáveis demográficas, socioeconômicas e aspectos relacionados ao acesso a esses medicamentos.

**MÉTODOS:**

Estudo transversal, analítico, baseado nos dados da Pesquisa Nacional sobre Acesso, Utilização e Promoção do Uso Racional de Medicamentos (PNAUM), de base populacional e amostra probabilística, realizada entre setembro/2013 e fevereiro/2014, em 20.404 domicílios urbanos brasileiros. A prevalência foi calculada a partir do relato das mulheres de 15 a 49 anos, não grávidas, sobre o uso de contraceptivos orais ou contraceptivos injetáveis. As variáveis independentes foram sexo, idade, escolaridade, nível socioeconômico, região geográfica e situação conjugal. Também foram analisados acesso, fontes de financiamento, fontes de obtenção e medicamentos citados. As análises estatísticas consideraram intervalos de confiança de 95% (IC95%) e teste Qui-quadrado de Pearson para avaliação da significância estatística das diferenças entre os grupos, considerando o nível de significância de 5%.

**RESULTADOS:**

A prevalência de uso de contraceptivos orais (CO) foi 28,2% e de contraceptivos injetáveis (CI), 4,5%. A prevalência de contraceptivos orais foi maior no Sul (37,5%) e menor no Norte (15,7%). Para contraceptivos injetáveis não houve diferença entre as regiões. O acesso foi maior para as usuárias de contraceptivos orais (90,7%) do que de contraceptivos injetáveis (81,2%), assim como o pagamento por desembolso direto (CO 78,1%; CI 58,0%). As usuárias que pagaram pelo contraceptivo compraram na farmácia comercial (CO 95,0% e CI 86,6%) e na Farmácia Popular (CO 4,8% e CI 12,7%). A principal fonte de obtenção gratuita foi o SUS (CO 86,7%; CI 96,0%). Amostra grátis foi citada por 10,4% das usuárias que não pagaram pelos contraceptivos orais. A maioria das usuárias que pagaram, não tentou obter no SUS. Contraceptivos orais combinados monofásico foram os mais citados (71,6%) e a combinação levonorgestrel+etinilestradiol de baixa concentração representou 38,7% destes. Os medicamentos mais citados constam na Relação Nacional de medicamentos Essenciais.

**CONCLUSÕES:**

A maioria das mulheres entre 15 e 49 anos que referiram usar contraceptivos obteve acesso ao medicamento, usa contraceptivos orais combinados monofásico, de eficácia e segurança adequada, obtido com pagamento do próprio bolso, principalmente, nas farmácias comerciais.

## INTRODUCTION

The International Conference on Population and Development (ICPD) held in Egypt (1994) is a milestone in defining the right to family planning^3^. In Brazil, the *Política Nacional de Direitos Sexuais e Direitos Reprodutivos* (National Policy for Sexual Rights and Reproductive Rights) and national policies related to women’s health^a^ strengthen the guarantee of constitutional rights related to family planning and establish government responsibilities^b^. These measures directly affect reproductive health and the improvement of socioeconomic indicators.

Information on the use of contraceptive methods helps policy management in this area. Use of contraceptive methods has increased worldwide, from 54.8% (95%CI 52.3–57.1) in 1990 to 63.3% (95%CI 60.4–66.0) in 2010.1 However, according to regional characteristics, the studies may have important methodological variations, especially regarding the characteristics of the study population (age of users, sexual activity, data source, etc.)^4^ ^,^ ^8^ ^,^ ^9^ ^,^ ^18^.

Studies in different countries show distinct regional patterns in the use of contraceptive methods. Short-term reversible methods are commonly used in Africa and Europe; long-term or permanent methods are used in Asia and North America. Latin America, the Caribbean and Oceania show a combination of different methods^7^.

The most common methods reported by adolescents are male condoms and oral contraceptives. Among women in their 20s, medium- and long-term reversible methods prevail (injectable contraceptives, implants and intra-uterine device). Female and male sterilization increase from the age of 30 onwards^14^ ^,^ ^21^.

Contraceptive use in Brazil was investigated in the *Pesquisa Nacional de Demografia e Saúde da Criança e da Mulher* (PNDS – National Survey on Children’s and Women’s Health and Demographics) in 1996 and 2006^14^. In the 2006 PNDS, 65.2% of women aged 15 to 49 reported using a contraceptive method deemed as modern. When traditional methods were included (fertility awareness, periodic abstinence, among others), prevalence was 67.8%. The most common were oral contraceptive (22.1%), female sterilization (21.8%), male condom (12.9%), injectable contraceptive (3.5%), and male sterilization (3.3%)^15^.

The Brazilian Ministry of Health funds and purchases contraceptives and inputs under *Programa Saúde da Mulher* (Women’s Health Program). Medicines supplied via public health services and *Programa Farmácia Popular do Brasil* (PFPB – Brazilian Popular Pharmacy Program) and included in the *Relação Nacional de Medicamentos Essenciais* (RENAME – National List of Essential Medicines) are: medroxyprogesterone acetate; norethisterone enanthate + estradiol valerate; ethinyl estradiol + levonorgestrel 0.03 mg + 0.15 mg; and norethindrone 0.35 mg. Public health services also supply emergency contraceptives: levonorgestrel 0.75 mg and misoprostol 0.025 mg and 0.2 mg^c^.

This study aimed to analyze the prevalence of current use of oral and injectable contraceptives by Brazilian women, according to demographic and socioeconomic variables and issues related to access to those medicines.

## METHODS

This cross-sectional descriptive study was based on data from the *Pesquisa Nacional sobre Acesso, Utilização e Promoção do Uso Racional de Medicamentos* (PNAUM – National Survey on Access, Use and Promotion of Rational Use of Medicines), carried out from September 2013 to February 2014, with a probability sampling of the population living in permanent private households in Brazilian urban areas. Data were collected via face-to-face interviews in 20,404 households, using questionnaires on electronic devices. The data are from a complex sample with national representation covering the five Brazilian regions, stratified by gender and age groups. The scope, sampling, and other methodological procedures of the survey, as well as the instruments used and aspects related to data collection, are available in the PNAUM methodology article^12^.

Two databases with different denominators were used for analysis. One comprises the sample, non-pregnant women aged 15 to 49 who answered the questionnaire block on contraceptives (12,364 valid interviews). The other relates to the medicines reported.

The independent variables were: age group (15-19, 20-29 and 30-49); socioeconomic classification of the *Associação Brasileira de Empresas de Pesquisa* –ABEP (Brazilian Association of Research Companies) (A/B, C and D/E) (http://www.abep.org); Brazilian region of residence (North; Northeast; Southeast; South; Midwest); level of education (0-8; 9-11; 12 or more years of schooling); and marital status (with partner; without partner).

Answers to the question “who indicated” were categorized as: medical indication; by other health professionals (pharmacist, nurse, other); self-administration (all other indications).

Prevalence of use of oral contraceptives (OC) was calculated for those who responded positively to the question: “Are you taking any birth control pill to prevent pregnancy?” For prevalence of use of injectable contraceptives (IC), the question was: “Are you taking any injection to prevent pregnancy?”

Prevalence of access was calculated from the answers to the questions: OC – “Did you miss taking the pill any day in the last month?” and IC – “Did you miss taking the injection for some time in the last year? If so, why?” “Yes” answers justified by “ran out of contraceptives” or “had no money to buy them” were counted as no access.

Calculation of OC access considered women who had not missed taking the contraceptive in the previous 30 days and those that had not used it for “health problems,” “forgot to take it,” “had no sexual activity,” “was in the interval between packets”, “there’s no need to take it every day.” Calculation of IC access considered women who had not missed taking the contraceptive in the previous year and those who had not used it for “health problems,” “forgot to take it,” “had no sexual activity,” “there’s no need to take it every month or quarter,” “wanted to get pregnant.”

Payment methods considered answers to the question, “Did you pay for this contraceptive yourself?” “Yes” answers were computed as direct payment; “no” answers were computed as free of charge access.

In the analysis of sources, those who paid were asked where the medicine was purchased (Popular Pharmacy Program, retail pharmacy, other). Those who obtained the medicine free of charge were asked where they obtained it (Brazilian Unified Health System – SUS, free sample, other). Users who paid were asked if they had tried to obtain it free of charge from SUS.

Respondents were asked to show the medicine packages, and, in the absence thereof, to report the contraceptive’s name. Active ingredients and their respective dosages were defined from the brand names. The analysis considered 3,009 medicines, 226 of which could not be identified (no packaging; unknown medicine name; brand names with more than one formulation; other categories of medicines; information recording problems). OC were classified as monophasic (MCOC), biphasic, and triphasic combinations, isolated progestogens and emergency pills. MCOC were classified according to estrogen levels: medium or high (≥ 0.05 mg); low (0.035; 0.03 and 0.02 mg) and ultralow (0.015 mg)^10^.

Ninety-five per cent confidence intervals (95%CI) were calculated. Pearson Chi-square test was used to evaluate the statistical significance of differences between the groups, considering a 5% significance level. All analyses were performed with the SPSS20.0 statistical package, using the CSPLAN command set suitable for the analysis of complex samples and ensuring the necessary weighting, according to the sample design.

Study limitations include lack of packaging, especially of injectable contraceptives, and of brand names, which restricts the identification of the most commonly used contraceptives. Regarding sources, references to the Popular Pharmacy Program may be uncertain, since the term “popular” can be part of the brand name of pharmaceutical establishments. The lack of further studies with the same scope of PNAUM prevents comparisons with previously published results.

The project was approved by the Brazilian National Committee for Ethics in Research (CONEP – Opinion 398.131, of September 16, 2013) and all interviews were conducted after the respondents had signed the informed consent form.

## RESULTS

Prevalence of OC and IC use among non-pregnant women aged 15-49, resident in Brazilian urban areas, was 32.7% (95%CI 31.1–34.4). Non-pregnant women who reported to be breastfeeding were 7.4% (95%IC 6.8–8.2), and of those, 42.6% (95%CI 37.6–47.8) reported using contraceptives, accounting for 6.6% (95%CI 5.9–7.4) of users.

Regarding indication for use, all IC users reported having medical indication. OC users reported medical indication (90.4%; 95%CI 88.7–91.9), self-administration (5.6%; 95%CI 4.6–6.8), and indication by other health professionals (2.5%; 95%CI 1.7–3.6).

Prevalence data considering age groups, Brazilian regions, ABEP categories, level of education, and marital status are shown in Table 1. OC use is higher compared to monthly or quarterly IC use, and both were more prevalent in the 20 to 29 age group. Prevalence of contraceptive use was higher in the South region and lower in the North region, reflecting the pattern of OC use. Regarding prevalence of IC use, there were no statistically significant differences between regions. Reported contraceptive use is similar in all socioeconomic and education categories. Regarding marital status, users who reported living with a partner showed a higher prevalence of use, for both OC and IC.

**Table 1:** Prevalence of use of oral and injectable contraceptives by women aged 15 to 49, excluding pregnant women, according to age, socioeconomic classa, Brazilian region, level of education and marital status. PNAUM, Brazil, 2014.

Variable	Oral contraceptive		Injectable contraceptive		Total	
		
	%	95%CI^b^	%	95%CI^b^	%	95%CI^b^
Age group (complete years)	p < 0.001		p < 0.001		p < 0.001	
15-19	20.2	16.0–25.3	3.7	2.2–6.2	23.9	19.3–29.2
20-29	40.7	38.1–43.3	8.8	7.3–10.5	49.5	46.9–52.0
30-49	23.6	21.9–25.5	2.5	2.0–3.2	26.2	24.3–28.1
Region	p < 0.001		p = 0.069		p < 0.001	
North	15.7	13.0–18.9	4.6	3.2–6.7	20.4	16.8–24.5
Northeast	23.6	21.3–26.0	5.8	4.5–7.4	29.4	26.8–32.1
Southeast	29.8	27.0–32.8	3.9	3.0–5.2	33.8	30.8–36.8
South	37.5	34.8–40.2	4.7	3.7–5.9	42.2	39.4–45.0
Midwest	29.7	26.8–32.8	3.4	2.4–4.7	33.1	30.3–36.1
Socioeconomic class^a^	p = 0.324		p = 0.089		p = 0.840	
A/B	30.1	27.0–33.4	3.3	2.5–4.5	33.5	30.4–36.6
C	27.6	25.9–29.5	4.8	4.0–5.8	32.4	30.5–34.4
D/E	27.4	24.3–30.8	5.1	3.8–6.7	32.5	29.2–36.0
Level of education	p = 0.415		p = 0.207		p = 0.901	
0 a 8 years of schooling	28.9	27.0–30.9	4.1	3.4–4.9	33.0	31.0–35.0
9 a 11 years of schooling	27.1	24.7–29.6	5.3	4.1–7.0	32.4	29.8–35.2
12 + years of schooling	27.5	23.8–31.6	4.6	3.2–6.8	32.2	28.4–36.2
Marital status	p < 0.001		p = 0.025		p < 0.001	
Partner	29.3	27.4–31.3	5.3	4.5–6.3	34.7	32.7–36.7
No partner	21.6	19.6–23.7	3.9	3.1–5.0	25.5	23.3–27.8

Total	28.2	26.6–29.8	4.5	3.9–5.2	32.7	31.1–34.4

^a^ According to *Critério de Classificação Econômica Brasil* 2013 (CCEB 2013 – Brazilian Economic Classification Criterion) of *Associação Brasileira de Empresas de Pesquisa* (ABEP – Brazilian Association of Survey Companies). Available from: www.abep.org

^b^ Percentages weighted by the sampling weights (sample not self-weighted).

Data on access and payment are shown in Table 2. About 90.0% of OC users said they did not miss taking contraceptives in the previous 30 days, and when they did, the reason was not related to access problems (financial or lack of medicines). Regional or socioeconomic class differences were not statistically significant. Most OC and IC users paid for the contraceptives, with higher prevalence for OC users compared to IC users. For OC users who paid for contraceptives, there were no differences between Brazilian regions; however, for IC users, most of them in the South and Southeast regions did not pay for the IC. Regarding socioeconomic status and payment, only OC users showed differences. In brackets A/B, prevalence of paying users was higher, while in brackets C/D it was lower.

**Table 2:** Prevalence of access to and direct payment of oral (OC) and injectable contraceptives (IC) in Brazil, per women aged 15-49 who reported using contraceptives, considering Brazilian region and socioeconomic classa. PNAUM, Brazil, 2014.

Variable	Access^b^				Direct payment^b^			
	
	OC		IC		OC		IC	
			
	%	95%CI	%	95%CI	%	95%CI	%	95%CI
Region	p = 0.060		p = 0.023		p = 0.623		p < 0.001	
North	92.6	88.9–95.2	82.8	74.0–89.0	81.2	70.6–88.6	85.1	71.1–92.9
Northeast	89.2	84.9–92.4	73.2	61.5–82.4	78.4	73.4–82.6	69.0	55.4–80.0
Southeast	91.6	88.2–94.1	88.8	79.2–94.2	76.7	70.5–82.0	48.7	36.3–61.2
South	92.5	89.4–94.7	75.4	63.2–84.5	78.3	73.6–82.4	38.0	25.9–51.8
Midwest	84.4	77.4–89.5	88.7	77.8–94.6	82.5	77.3–86.7	69.9	49.4–84.6
ABEP	p = 0.788		p = 0.479		p = 0.005		p = 0.841	
A/B	90.2	86.3–93.0	86.9	75.7–93.5	84.5	79.1–88.6	62.5	46.6–76.1
C	90.5	88.0–92.5	79.1	70.9–85.5	77.4	73.6–80.8	57.3	47.9–66.2
D/E	91.7	87.9–94.3	82.1	69.9–90.1	71.3	63.8–77.8	57.5	43.6–70.3

Total	90.7	88.8–92.3	81.2	75.6–85.8	78.1	74.9–81.0	58.0	50.7–64.9

^a^ According to *Critério de Classificação Econômica Brasil* 2013 (CCEB 2013 – Brazilian Economic Classification Criterion) of *Associação Brasileira de Empresas de Pesquisa* (ABEP – Brazilian Association of Survey Companies). Available from: www.abep.org

^b^ Percentages weighted by the sampling weights (sample not self-weighted).

**Figure 1:**
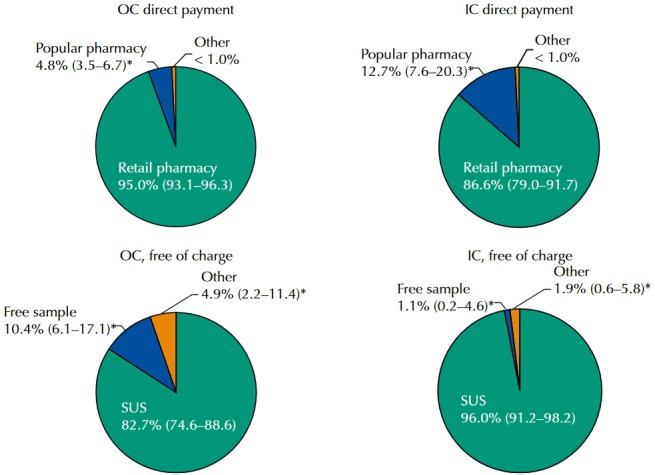
Sources of oral (OC) and injectable (IC) contraceptives, according to means of payment (direct payment or free of charge). PNAUM, Brazil, 2014.

Figure 1 shows the sources of OC and lC regarding payment. Retail pharmacies were the main source for paid contraceptives. The Popular Pharmacy Program was an important source for the purchase of IC (12.7%; 95%CI 7.6–20.3), while for OC, references to Popular Pharmacy Program were less significant (4.8%, 95%CI 5.5–6.7). SUS was the most reported source for free of charge contraceptives, especially by IC users (96.0%, 95%CI 91.2–98.2). However, free samples were reported by 10.4% (95%CI 6.1–17.1) of OC users who did not pay for the medicine.

* Percentages weighted by the sampling weights (sample not self-weighted).

**Figure 2:**
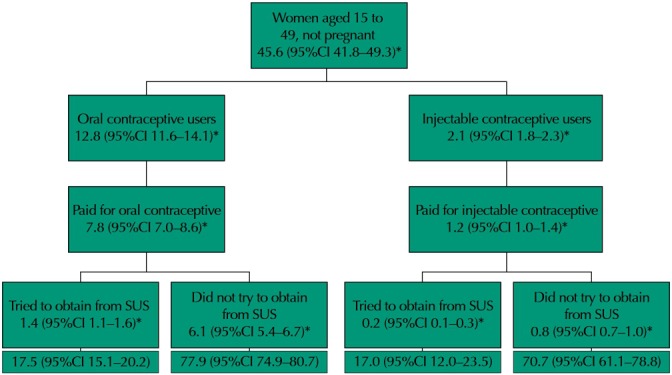
Attempt to obtain oral and injectable contraceptives from the Brazilian Unified Health System (SUS) by users who reported having paid for the medicine.

Paying users were asked if they had tried to obtain the medicine from the public health system (SUS). Data are shown in Figure 2. Most of the OC and IC users did not try to obtain them from SUS. OC and IC users who reported having tried to obtain the medicine from SUS accounted for, respectively, 17.5% (95%CI 15.1–20.2) and 17.0% (95%CI 12.0–23.5) of users who paid for the contraceptive. That represents approximately 1.6 million women.

* Estimate of the number of women in the urban population, in millions

Packages were shown by 63.0% (95%CI 59.8–66.2) of OC users and 22.3% (95%CI 17.5–28.1) of IC users. The reported products are shown in Table 3. Unidentified contraceptives accounted for 7.6% (95%CI 6.2–9.5) of the total. Prevalence of MCOC use was higher, 71.6% (95%CI 68.9–74.1), and of those, most users reported the use of combinations with low-level estrogen. Combinations with estrogen levels above 0.05 mg were reported by 3.5% (95%CI 2.7–4.5) of MCOC users. The most commonly reported MCOC and injectable combinations are listed in RENAME. Regarding oral contraceptives with isolated progestogen, the most frequently reported medicine was desogestrel, which is not listed in RENAME.

**Table 3:** Main oral and injectable contraceptives (> 0.5% prevalence) used by women of childbearing age. PNAUM, Brazil, 2014.

Main contraceptives	%^a^	95%CI^a^
Monophasic combined oral	71.6	68.9–74.1
Levonorgestrel + ethinyl estradiol (low level)^b,c^	38.7	35.3–42.1
Cyproterone + ethinyl estradiol (low level)^b^	9.2	7.8–10.8
Gestodene + ethinyl estradiol (low level)^b^	8.1	6.8–9.7
Drospirenone + ethinyl estradiol (level)^b^	5.8	4.4–7.5
Levonorgestrel + ethinyl estradiol (medium or high level)^b^	3.5	2.7–4.5
Gestodene + ethinyl estradiol (ultralow level)^b^	3.2	2.2–4.6
Desogestrel + ethinyl estradiol (low level)^b^	2.6	1.9–3.7
Bi- or triphasic combined oral	3.2	2.4–4.3
Ethinylestradiol + levonorgestrel	1.9	1.3–2.8
Estradiol valerate + dienogest	0.8	0.4–1.7
Oral with isolated progestogen	5.0	3.9–6.4
Desogestrel	3.3	2.4–4.6
Norethisterone acetate^c^	1.6	1.1–2.3
Injectable	12.6	10.7–14.8
Norethisterone enanthate + valerate estradiol^c^	4.4	3.4–5.7
Medroxyprogesterone acetate^c^	2.9	2.1–4.0
Non-identified	7.6	6.2–9.5

^a^ Percentages weighted by the sampling weights (sample not self-weighted).

^b^ ultralow (≤ 0.015 mg of estrogen); low (0.035, 0.03, 0.02 mg of estrogen) and medium and high (≥ 0.05 mg of estrogen).

^c^ Contraceptives listed in *Relação Nacional de Medicamentos Essenciais* (RENAME – National List of Essential Medicines) and available at Popular Pharmacy Program.

## DISCUSSION

The study investigated the reported use of oral and injectable contraceptives among non-pregnant women aged 15 to 49, sexually active or not.

Prevalence of use was 28.2% (95%CI 26.6–29.8) for OC and 4.5% (95%CI 3.9–5.2) for IC, and most women reported using contraceptives by medical indication. OC prevalence was higher in the South region and lower in the North region. Access was higher for OC users compared to IC users. Most users reported paying for contraceptives, with a significant difference between OC and IC users and between geographical regions. In the South and Southeast regions, most IC users did not pay for the medicines. Paying users bought them at retail pharmacies and Popular Pharmacy Program, which was more often used by IC users than OC users. Most paying users did not try to obtain them from SUS. The main source of free of charge contraceptives was SUS, and 10.4% of OC users reported using free samples. The most cited contraceptives are listed in RENAME, with higher prevalence of MCOC, the most prevalent of which was the levonorgestrel + ethinyl estradiol combination with low-level estrogen (38.7% of MCOC).

Report of medical indication for contraceptive use was high; however, the indication may have occurred at any time in the past, with the same prescription being used repeatedly.

Overall prevalence of OC use was higher than the overall figure for Latin America and the Caribbean (24.0%) and similar to that of South America (29.0%), European countries (30.0%), and the US (28.0%)^1^ ^,^ ^4^ ^,^ ^7^ ^-^ ^9^ ^,^ ^18^. For IC, prevalence was lower than that reported for injectable contraceptives and implants in South America (9.0%)^1^.

The PNDS analyzed prevalence of use among all women, women living with a partner, and sexually active women living without a partner. Data from this study show that current prevalence of OC and IC use among women living without a partner (21.6% and 3.9%) is similar to prevalence for all women in 2006 (22.1% and 3.5%). For those who reported living with a partner, prevalence was 29.3% for OC and 5.3% for IC, higher than the 2006 figures (24.7% and 4.0%, respectively), and close to those found for sexually active women without a partner (30.3% and 4.4%, respectively). The increase in prevalence of current use, for both OC and IC, is consistent with the trend observed in the comparison between the 1996 and 2006 PNDS, a period that showed a significant reduction in female sterilization^14^ ^,^ ^15^.

Use by age group is also similar to the pattern observed in the 2006 PNDS. Between ages 15 and 20, data from the 2006 PNDS show that 44.2% of young women had never engaged in sexual intercourse; however, 24.8% became pregnant before the age of 20, which makes contraception in this age group a public health issue^2^. Studies indicate advantages in the use of medium- and long-term reversible methods, including injectable contraceptives, especially in adolescence^5^ ^,^ ^11^. However, prevalence of CI use is low in all age groups, despite being significantly higher in the 20 to 29 age group.

Profile of use per region, as well as of access and payment, showed differences between OC and IC users, but as prevalence of OC use is much higher than IC use, it influences the total.

The lower prevalence of OC use in the North region is similar to data from the 2006 PNDS for women with partners^14^. One hypothesis is age composition, which is younger in the region, with an average of 22 years in the 2010 Census^22^. In the 2006 PNDS, the North region showed a high prevalence of female sterilization (41.0%), which may also contribute to the lower prevalence of OC use in the region.

Analysis of the variable payment by socioeconomic class shows that prevalence of free of charge access is higher in brackets D/E. This fact, coupled with the fact that the North and Northeast regions have a higher prevalence of unmet need for contraceptive methods^20^, points to the importance of viewing those regions in different ways regarding family planning.

No differences in prevalence of use were found for IC users between regions. However, payment and access showed marked regional differences. While most users in the North region paid for the medicines (87.7%), in the South region most obtained them free of charge (62.5%). On the other hand, access was significantly lower in the Northeast and South regions. This fact seems not to be influenced by socioeconomic class. The differences may be related to prescription profile or health service organization, which could not be investigated in this study.

Regarding sources of medicines, retail pharmacies are still the main site for the purchase of contraceptives^14^. The Brazilian Popular Pharmacy Program was introduced in 2004 with its own pharmacy network and expanded in 2006 to the retail pharmacy network, called “*Aqui Tem Farmácia Popular*” (Popular Pharmacy Here). In 2004-2012, there was a significant increase in the number of accredited units (750.0%) and municipalities covered by the program (528.0%)^17^. Popular Pharmacy Program has been reported in some studies as an alternative due to the ready availability of medicines and prompt service, especially when the supply of medicines in public network pharmacies is irregular^6^ ^,^ ^16^.

The main source of free of charge access was SUS; however, 10.4% of users who did not pay for OC reported using free samples. The Brazilian legislation provides that free samples of contraceptives must contain 100% of the amount of formulation registered with ANVISA, equivalent to one month of treatment. However, that does not guarantee access and effectiveness^19^.

Most paying users paid did not try to obtain contraceptives from SUS, suggesting that the population has not yet grasped the universal nature of the system. However, the number of users who paid for contraceptives but tried to obtain them from SUS is important, as it represents a need unfulfilled by the public service and that is often disregarded in medicine programming. About 1.4 million OC users and 200,000 IC users reported having tried to obtain medicines from SUS, accounting for 17.5% and 17.0% of OC and IC users, respectively, who paid for the contraceptive.

MCOC were the most frequently reported contraceptives, corroborating other studies^3^ ^,^ ^4^ ^,^ ^8^ ^,^ ^9^ ^,^ ^18^. These combinations have similar efficacy and the differences between formulations involve cardiovascular risks related to hormone levels, especially estrogen^10^. Most of the reported MCOC had low estrogen levels, with fewer risks of cardiovascular and thromboembolic phenomena. The advantages of the 0.02 mg level compared to the 0.03 and 0,035 mg levels, as well as to combinations with ultralow levels, remain controversial^10^. On the other hand, 3.5% of users reported contraceptives with high estrogen levels, which have increased cardiovascular risk and require the attention of health services. Overall, the combinations listed in RENAME were the most prevalent in all contraceptive sources.

In conclusion, most women aged 15 to 49 who reported using contraceptives had access to the medicine and use MCOC of appropriate efficacy and safety acquired by direct payment, mainly at retail pharmacies.

In public services, planning, procurement and distribution logistics of those medicines is essential to prevent shortages and ensure access. Spreading information on contraceptive options among prescribers and on sources of access is also essential in a universal system.

In recent years, significant investments have been made to improve the population’s access to medicines and the quality of pharmaceutical services. The regional characteristics shown in this study suggest differences in implementing public policies. Therefore, the results may contribute to improve free access to contraceptives by the population and reduce regional differences. Moreover, it is necessary to make progress in coordinating care and management in pharmaceutical services to ensure access to and adequate use of contraceptives, minimizing side effects and contraceptive failure, which is strongly related to inadequate use^13^.

## Footnotes

a Brasil. Lei nº 9.263, de 12 de janeiro de 1996. Regula o § 7º do art. 226 da Constituição Federal, que trata do planejamento familiar, estabelece penalidades e dá outras providências. *Diario Oficial da Uniao*, v.134, n.10, 15 jan. 1996. Seção 1

b Brasil. Constituição (1988). Constituição da República Federativa do Brasil. Brasília (DF): Senado; 1988.

c Ministério da Saúde. Portal da Saúde. Assistência farmacêutica na saúde da mulher. Brasília (DF): Ministério da Saúde; 2016 [cited 2016 Sept 14]. Available from: http://portalsaude.saude.gov.br/index.php/o-ministerio/principal/leia-mais-oministerio/1058-sctie-raiz/daf-raiz/componente-basicoda-assistencia-farmaceutica/l4-componente-basico-daassistencia-farmaceutica/15078-saude-da-mulher
